# Crystal structure of 1-(4-formyl­benzyl­idene)thio­semicarbazone

**DOI:** 10.1107/S1600536814017255

**Published:** 2014-08-06

**Authors:** Rosa Carballo, Arantxa Pino-Cuevas, Ezequiel M. Vázquez-López

**Affiliations:** a Departamento de Química Inorgánica, Facultade de Química, Edificio de Ciencias Experimentais, Universidade de Vigo, E-36310 Vigo, Galicia, Spain

**Keywords:** crystal structure, thio­semicarbazone, hydrogen bonds

## Abstract

The asymmetric unit of the title compound, C_9_H_9_N_3_OS, contains two approximately planar mol­ecules (r.m.s. deviations for 14 non-H atoms = 0.094 and 0.045 Å), with different conformations. In one of them, the C=O group is *syn* to the S atom and in the other it is *anti*. Each mol­ecule features an intra­molecular N—H⋯N hydrogen bond, which generates an *S*(5) ring. In the crystal, mol­ecules are linked by N—H⋯O and N—H⋯S hydrogen bonds, generating discrete networks; the *syn* mol­ecules form [010] chains and the *anti* mol­ecules form (100) sheets.

## Related literature   

For further synthetic details, see: Jagst *et al.* (2005 ▶). For structure–biological activity relationships in thio­semicarbazones, see: Lukmantara *et al.* (2013 ▶). For their biological properties, see: Serda *et al.* (2012 ▶).
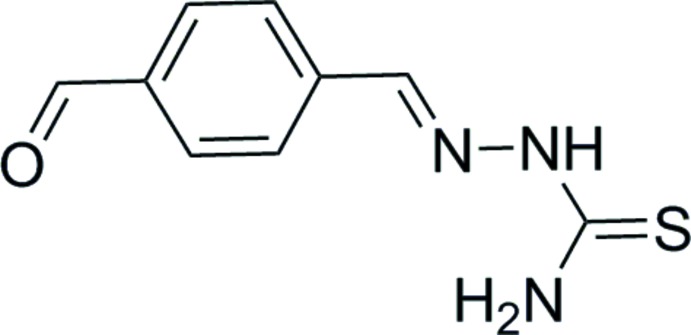


## Experimental   

### Crystal data  

C_9_H_9_N_3_OS
*M* *_r_* = 207.25
Monoclinic, 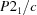

*a* = 12.3888 (9) Å
*b* = 11.7972 (8) Å
*c* = 14.9428 (11) Å
β = 110.286 (1)°
*V* = 2048.5 (3) Å^3^
*Z* = 8
Mo *K*α radiation
μ = 0.29 mm^−1^
*T* = 293 K
0.51 × 0.44 × 0.33 mm

### Data collection  

Bruker SMART 1000 CCD diffractometer
Absorption correction: multi-scan (*SADABS*; Sheldrick, 1996 ▶) *T*_min_ = 0.693, *T*_max_ = 0.746
19018 measured reflections
4920 independent reflections
3344 reflections with *I* > 2σ(*I*)
*R*_int_ = 0.022

### Refinement  

*R*[*F* ^2^ > 2σ(*F* ^2^)] = 0.040
*wR*(*F* ^2^) = 0.119
*S* = 1.03
4920 reflections
277 parameters
H atoms treated by a mixture of independent and constrained refinement
Δρ_max_ = 0.36 e Å^−3^
Δρ_min_ = −0.35 e Å^−3^

### 

Data collection: *SMART* (Bruker, 2008 ▶); cell refinement: *SAINT* (Bruker, 2008 ▶); data reduction: *SAINT*; program(s) used to solve structure: *SHELXS2013* (Sheldrick, 2008 ▶); program(s) used to refine structure: *SHELXL2013* (Sheldrick, 2008 ▶); molecular graphics: *Mercury* (Macrae *et al.*, 2008 ▶); software used to prepare material for publication: *publCIF* (Westrip, 2010 ▶).

## Supplementary Material

Crystal structure: contains datablock(s) I, New_Global_Publ_Block. DOI: 10.1107/S1600536814017255/hb7254sup1.cif

Structure factors: contains datablock(s) I. DOI: 10.1107/S1600536814017255/hb7254Isup2.hkl

Supporting information file. DOI: 10.1107/S1600536814017255/hb7254Isup3.cml

**Fig. 1.**
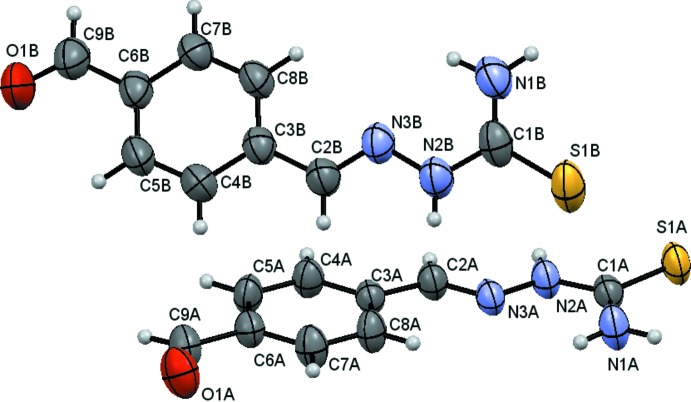
ORTEP view of the two molecules of the title compound. Displacement ellipsoids shown at the 50% probability level.

ORTEP . DOI: 10.1107/S1600536814017255/hb7254fig1.tif
*ORTEP* view of the two mol­ecules of the title compound. Displacement ellipsoids shown at the 50% probability level.

**Fig. 2.**
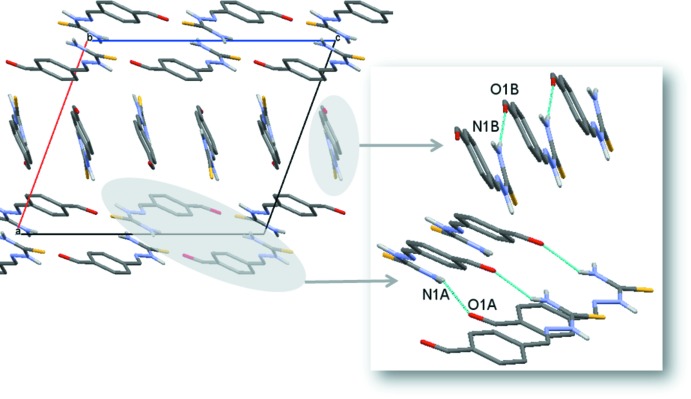
View of the crystal packing showing the two different chains.

. DOI: 10.1107/S1600536814017255/hb7254fig2.tif
View of the crystal packing showing the two different chains.

CCDC reference: 1016158

Additional supporting information:  crystallographic information; 3D view; checkCIF report

## Figures and Tables

**Table 1:** Hydrogen-bond geometry (Å, °)

*D*—H⋯*A*	*D*—H	H⋯*A*	*D*⋯*A*	*D*—H⋯*A*
N1*A*—H1*NA*⋯N3*A*	0.84 (3)	2.32 (2)	2.630 (2)	102.0 (19)
N1*A*—H1*NA*⋯O1*A* ^i^	0.84 (3)	2.41 (3)	3.190 (3)	154 (2)
N1*A*—H2*NA*⋯S1*A* ^ii^	0.87 (3)	2.52 (3)	3.391 (2)	172 (2)
N2*A*—H3*NA*⋯S1*B* ^iii^	0.84 (2)	2.50 (2)	3.3270 (19)	166.1 (19)
N1*B*—H1*NB*⋯N3*B*	0.91 (3)	2.21 (3)	2.619 (3)	106 (3)
N1*B*—H2*NB*⋯O1*B* ^iv^	0.88 (3)	2.01 (3)	2.857 (3)	161 (3)
N2*B*—H3*NB*⋯S1*A* ^v^	0.84 (2)	2.58 (2)	3.409 (2)	171 (2)

Symmetry codes: (i) 
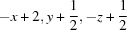
; (ii) 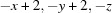; (iii) 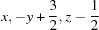; (iv) 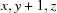; (v) 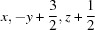.

## supplementary crystallographic information

### S1. Chemical context

The study of the thio­semicarbazones is inter­esting because they are compounds which show diverse biological properties (Serda *et al.*, 2012) and pharmacological activities (Lukmantara *et al.*, 2013). Also the thio­semicarbazones are of inter­est from a supra­molecular point of view since they can be functionalized to give different supra­molecular arrays by hydrogen bonds.

### S2. Structural commentary

We report here the synthesis and structural characterization of (4-formyl­benzyl­idine)-thio­semicarbazone (Fig.1). The two molecules in the asymmetric unit are structurally different due to the different orientation of the carbonyl group respect to the thio­semicarbazone chain. The thio­semicarbazone moiety in both molecules shows an E conformation with the sulfur atom trans to the iminic nitro­gen N3 atom. The molecules labeled as B are linked into lineal chains by N—H···O hydrogen bonds with a d(N···O) of 2.857 (3) Å but the molecules labeled as A use the same kind of hydrogen bond with a longer d(N···O) of 3.190 (3) Å to form helical chains (Fig. 2). The two types of chains are packed by N—H···S hydrogen bonds with d(N—S) in the range 3.32-3.41 Å and (NHS) angles close to linearity (between 166 and 172°).

### S5. Synthesis and crystallization

A solution of thio­semicarbazide (342mg, 3.72 mmol) in 50 ml of water was slowly added at 50°C to a solution of terephthaldicarboxaldehyde (500 mg, 3.73 mmol) in 100 ml water. Then the mixture was stirred at 50°C for 30 mins. Once cooled to room temperature, the yellow solid was filtered off and vacuum dried. Yellow prisms were obtained by recrystallization from EtOH/H_2_O (1:1) solution. Yield: 78%. M.pt: 212–214°C. IR data (KBr, cm^-1^): 3452w, 3328m, 3152m ν(NH); 2974w, 2863w ν(C—H aldehyde); 1686s ν(C=O); 1533s, 1281m ν(C=N), 830m, 793m ν(C=S). ^1^H NMR data (DMSO-d_6_, ppm): 10.60 (s, 1H, N(2)—H); 10.01 (s, 1H, C(1)—H); 8.32 (s, 1H, N(2)—H); 8.15 (s, 1H, N(2)—H); 8.09 (s, 1H, C(8)—H); 8.02 (d, 2H, J = 8.2 Hz, C(3,7)-H); 7.91 (d, 2H, J = 8.2 Hz, C(4,6)-H).

### Crystal data

**Table:**

C_9_H_9_N_3_OS	*F*(000) = 864
*M**_r_* = 207.25	*D*_x_ = 1.344 Mg m^−^^3^
Monoclinic, *P*2_1_/*c*	Mo *K*α radiation, λ = 0.71073 Å
*a* = 12.3888 (9) Å	Cell parameters from 6097 reflections
*b* = 11.7972 (8) Å	θ = 2.3–27.2°
*c* = 14.9428 (11) Å	µ = 0.29 mm^−^^1^
β = 110.286 (1)°	*T* = 293 K
*V* = 2048.5 (3) Å^3^	Prism, yellow
*Z* = 8	0.51 × 0.44 × 0.33 mm

### Data collection

**Table:**

Bruker SMART 1000 CCD diffractometer	3344 reflections with *I* > 2σ(*I*)
Radiation source: sealed X-ray tube	*R*_int_ = 0.022
φ and ω scans	θ_max_ = 28.1°, θ_min_ = 1.8°
Absorption correction: multi-scan (*SADABS*; Sheldrick, 1996)	*h* = −16→16
*T*_min_ = 0.693, *T*_max_ = 0.746	*k* = −15→15
19018 measured reflections	*l* = −19→19
4920 independent reflections	

### Refinement

**Table:**

Refinement on *F*^2^	0 restraints
Least-squares matrix: full	Hydrogen site location: mixed
*R*[*F*^2^ > 2σ(*F*^2^)] = 0.040	H atoms treated by a mixture of independent and constrained refinement
*wR*(*F*^2^) = 0.119	*w* = 1/[σ^2^(*F*_o_^2^) + (0.0442*P*)^2^ + 0.8755*P*] where *P* = (*F*_o_^2^ + 2*F*_c_^2^)/3
*S* = 1.03	(Δ/σ)_max_ = 0.001
4920 reflections	Δρ_max_ = 0.36 e Å^−^^3^
277 parameters	Δρ_min_ = −0.35 e Å^−^^3^

### Special details

**Table:**

Geometry. All e.s.d.'s (except the e.s.d. in the dihedral angle between two l.s. planes) are estimated using the full covariance matrix. The cell e.s.d.'s are taken into account individually in the estimation of e.s.d.'s in distances, angles and torsion angles; correlations between e.s.d.'s in cell parameters are only used when they are defined by crystal symmetry. An approximate (isotropic) treatment of cell e.s.d.'s is used for estimating e.s.d.'s involving l.s. planes.

### Fractional atomic coordinates and isotropic or equivalent isotropic displacement parameters (Å^2^)

**Table:**

	*x*	*y*	*z*	*U*_iso_*/*U*_eq_	
N3A	0.89210 (14)	0.63007 (12)	−0.01356 (11)	0.0479 (4)	
S1A	0.90693 (5)	0.91696 (4)	−0.13892 (4)	0.06120 (17)	
O1A	0.89088 (17)	0.16790 (14)	0.29083 (12)	0.0778 (5)	
N1A	0.97843 (19)	0.83357 (16)	0.03697 (14)	0.0646 (5)	
C1A	0.92577 (17)	0.81529 (15)	−0.05446 (14)	0.0488 (4)	
N2A	0.88481 (15)	0.71091 (13)	−0.08158 (13)	0.0519 (4)	
C2A	0.84890 (17)	0.53375 (15)	−0.04474 (14)	0.0499 (4)	
H2A	0.8172	0.5214	−0.1102	0.060*	
C3A	0.84875 (16)	0.44243 (14)	0.02136 (13)	0.0446 (4)	
C4A	0.80002 (18)	0.33905 (16)	−0.01569 (14)	0.0536 (5)	
H4A	0.7659	0.3300	−0.0813	0.064*	
C5A	0.80199 (18)	0.24931 (15)	0.04467 (14)	0.0549 (5)	
H5A	0.7691	0.1803	0.0195	0.066*	
C6A	0.85265 (17)	0.26217 (15)	0.14207 (14)	0.0487 (4)	
C7A	0.9008 (2)	0.36573 (16)	0.17931 (14)	0.0576 (5)	
H7A	0.9348	0.3746	0.2450	0.069*	
C8A	0.89848 (19)	0.45524 (16)	0.11987 (14)	0.0550 (5)	
H8A	0.9302	0.5245	0.1455	0.066*	
C9A	0.8559 (2)	0.16499 (18)	0.20519 (17)	0.0615 (5)	
H9A	0.8286	0.0959	0.1763	0.074*	
H1NA	0.990 (2)	0.782 (2)	0.0779 (17)	0.069 (7)*	
H2NA	1.007 (2)	0.900 (2)	0.0570 (17)	0.075 (7)*	
H3NA	0.8488 (18)	0.6956 (18)	−0.1392 (16)	0.054 (6)*	
S1B	0.70719 (7)	0.87078 (5)	0.20508 (5)	0.0836 (2)	
O1B	0.36864 (17)	−0.02352 (14)	0.06781 (17)	0.1027 (7)	
N1B	0.5055 (2)	0.77556 (19)	0.11523 (17)	0.0728 (6)	
C1B	0.6141 (2)	0.76241 (17)	0.16872 (15)	0.0633 (6)	
N2B	0.6506 (2)	0.65472 (15)	0.19199 (15)	0.0659 (5)	
C2B	0.61285 (19)	0.46749 (17)	0.18659 (16)	0.0596 (5)	
H2B	0.6866	0.4587	0.2310	0.072*	
N3B	0.57577 (16)	0.56654 (14)	0.15795 (13)	0.0591 (4)	
C3B	0.54146 (18)	0.36782 (16)	0.15082 (15)	0.0539 (5)	
C4B	0.5824 (2)	0.26093 (18)	0.18761 (17)	0.0636 (6)	
H4B	0.6538	0.2549	0.2358	0.076*	
C5B	0.5189 (2)	0.16464 (18)	0.15366 (18)	0.0663 (6)	
H5B	0.5477	0.0941	0.1784	0.080*	
C6B	0.41212 (19)	0.17280 (17)	0.08275 (16)	0.0574 (5)	
C7B	0.37016 (19)	0.27858 (17)	0.04567 (16)	0.0592 (5)	
H6B	0.2983	0.2843	−0.0020	0.071*	
C8B	0.43420 (19)	0.37484 (17)	0.07894 (16)	0.0583 (5)	
H7B	0.4056	0.4451	0.0532	0.070*	
C9B	0.3412 (2)	0.07213 (19)	0.0444 (2)	0.0723 (6)	
H9B	0.2691	0.0838	−0.0017	0.087*	
H1NB	0.463 (3)	0.712 (3)	0.093 (2)	0.107 (10)*	
H2NB	0.478 (2)	0.844 (2)	0.1003 (19)	0.085 (8)*	
H3NB	0.717 (2)	0.642 (2)	0.2304 (17)	0.065 (7)*	

### Atomic displacement parameters (Å^2^)

**Table:**

	*U*^11^	*U*^22^	*U*^33^	*U*^12^	*U*^13^	*U*^23^
N3A	0.0572 (9)	0.0343 (7)	0.0505 (8)	0.0015 (7)	0.0167 (7)	0.0081 (6)
S1A	0.0797 (4)	0.0362 (2)	0.0594 (3)	−0.0022 (2)	0.0134 (3)	0.0123 (2)
O1A	0.1133 (14)	0.0633 (10)	0.0600 (10)	0.0069 (9)	0.0341 (9)	0.0169 (8)
N1A	0.0936 (15)	0.0363 (9)	0.0541 (10)	−0.0053 (9)	0.0130 (10)	0.0050 (8)
C1A	0.0554 (11)	0.0351 (9)	0.0545 (11)	0.0034 (8)	0.0174 (9)	0.0043 (8)
N2A	0.0682 (11)	0.0343 (7)	0.0479 (9)	−0.0020 (7)	0.0135 (8)	0.0067 (7)
C2A	0.0602 (11)	0.0375 (9)	0.0477 (10)	−0.0014 (8)	0.0131 (8)	0.0043 (8)
C3A	0.0498 (10)	0.0347 (8)	0.0480 (10)	0.0006 (7)	0.0151 (8)	0.0032 (7)
C4A	0.0647 (12)	0.0430 (9)	0.0447 (10)	−0.0084 (9)	0.0082 (9)	0.0010 (8)
C5A	0.0638 (12)	0.0367 (9)	0.0586 (12)	−0.0116 (8)	0.0141 (10)	−0.0017 (8)
C6A	0.0576 (11)	0.0383 (9)	0.0507 (10)	−0.0004 (8)	0.0193 (9)	0.0057 (8)
C7A	0.0817 (15)	0.0445 (10)	0.0439 (10)	−0.0040 (10)	0.0183 (10)	−0.0002 (8)
C8A	0.0774 (14)	0.0351 (9)	0.0498 (10)	−0.0068 (9)	0.0187 (10)	−0.0045 (8)
C9A	0.0775 (15)	0.0449 (10)	0.0641 (13)	−0.0005 (10)	0.0271 (11)	0.0082 (9)
S1B	0.1164 (6)	0.0470 (3)	0.0657 (4)	−0.0135 (3)	0.0042 (3)	0.0020 (3)
O1B	0.0916 (13)	0.0436 (9)	0.158 (2)	−0.0036 (9)	0.0239 (13)	−0.0026 (11)
N1B	0.0818 (15)	0.0507 (11)	0.0826 (14)	0.0090 (11)	0.0243 (12)	0.0030 (11)
C1B	0.0916 (17)	0.0453 (11)	0.0517 (11)	0.0007 (11)	0.0233 (11)	−0.0012 (9)
N2B	0.0739 (13)	0.0445 (9)	0.0669 (12)	−0.0017 (9)	0.0088 (10)	−0.0003 (8)
C2B	0.0632 (13)	0.0471 (11)	0.0647 (13)	0.0019 (10)	0.0172 (10)	0.0008 (9)
N3B	0.0685 (11)	0.0429 (9)	0.0635 (10)	−0.0031 (8)	0.0199 (9)	−0.0036 (8)
C3B	0.0603 (12)	0.0434 (10)	0.0616 (12)	0.0037 (9)	0.0259 (10)	−0.0003 (9)
C4B	0.0605 (13)	0.0517 (11)	0.0738 (14)	0.0066 (10)	0.0172 (11)	0.0103 (10)
C5B	0.0705 (15)	0.0419 (10)	0.0884 (16)	0.0083 (10)	0.0299 (13)	0.0111 (10)
C6B	0.0607 (13)	0.0434 (10)	0.0742 (14)	0.0031 (9)	0.0312 (11)	−0.0022 (9)
C7B	0.0583 (12)	0.0483 (11)	0.0697 (13)	0.0068 (9)	0.0207 (10)	−0.0036 (10)
C8B	0.0650 (13)	0.0423 (10)	0.0672 (13)	0.0103 (9)	0.0225 (11)	0.0015 (9)
C9B	0.0709 (15)	0.0517 (12)	0.0965 (18)	−0.0014 (11)	0.0320 (13)	−0.0068 (12)

### Geometric parameters (Å, º)

**Table:**

N3A—C2A	1.274 (2)	S1B—C1B	1.681 (2)
N3A—N2A	1.374 (2)	O1B—C9B	1.195 (3)
S1A—C1A	1.6976 (18)	N1B—C1B	1.314 (3)
O1A—C9A	1.201 (3)	N1B—H1NB	0.91 (3)
N1A—C1A	1.312 (3)	N1B—H2NB	0.88 (3)
N1A—H1NA	0.84 (3)	C1B—N2B	1.353 (3)
N1A—H2NA	0.87 (3)	N2B—N3B	1.369 (2)
C1A—N2A	1.340 (2)	N2B—H3NB	0.84 (2)
N2A—H3NA	0.84 (2)	C2B—N3B	1.274 (3)
C2A—C3A	1.462 (2)	C2B—C3B	1.457 (3)
C2A—H2A	0.9300	C2B—H2B	0.9300
C3A—C4A	1.388 (2)	C3B—C8B	1.392 (3)
C3A—C8A	1.393 (3)	C3B—C4B	1.399 (3)
C4A—C5A	1.386 (3)	C4B—C5B	1.375 (3)
C4A—H4A	0.9300	C4B—H4B	0.9300
C5A—C6A	1.379 (3)	C5B—C6B	1.382 (3)
C5A—H5A	0.9300	C5B—H5B	0.9300
C6A—C7A	1.388 (3)	C6B—C7B	1.391 (3)
C6A—C9A	1.476 (3)	C6B—C9B	1.470 (3)
C7A—C8A	1.374 (3)	C7B—C8B	1.376 (3)
C7A—H7A	0.9300	C7B—H6B	0.9300
C8A—H8A	0.9300	C8B—H7B	0.9300
C9A—H9A	0.9300	C9B—H9B	0.9300
			
C2A—N3A—N2A	115.92 (16)	C1B—N1B—H1NB	118 (2)
C1A—N1A—H1NA	122.5 (16)	C1B—N1B—H2NB	119.2 (18)
C1A—N1A—H2NA	120.1 (16)	H1NB—N1B—H2NB	122 (3)
H1NA—N1A—H2NA	117 (2)	N1B—C1B—N2B	116.6 (2)
N1A—C1A—N2A	117.74 (17)	N1B—C1B—S1B	123.36 (18)
N1A—C1A—S1A	123.28 (15)	N2B—C1B—S1B	120.0 (2)
N2A—C1A—S1A	118.98 (15)	C1B—N2B—N3B	119.7 (2)
C1A—N2A—N3A	119.56 (17)	C1B—N2B—H3NB	120.4 (17)
C1A—N2A—H3NA	121.2 (15)	N3B—N2B—H3NB	119.7 (16)
N3A—N2A—H3NA	119.0 (15)	N3B—C2B—C3B	121.0 (2)
N3A—C2A—C3A	120.60 (17)	N3B—C2B—H2B	119.5
N3A—C2A—H2A	119.7	C3B—C2B—H2B	119.5
C3A—C2A—H2A	119.7	C2B—N3B—N2B	116.96 (19)
C4A—C3A—C8A	119.25 (16)	C8B—C3B—C4B	118.45 (19)
C4A—C3A—C2A	118.70 (17)	C8B—C3B—C2B	122.07 (18)
C8A—C3A—C2A	122.03 (16)	C4B—C3B—C2B	119.5 (2)
C5A—C4A—C3A	120.31 (17)	C5B—C4B—C3B	121.1 (2)
C5A—C4A—H4A	119.8	C5B—C4B—H4B	119.5
C3A—C4A—H4A	119.8	C3B—C4B—H4B	119.5
C6A—C5A—C4A	120.11 (17)	C4B—C5B—C6B	119.92 (19)
C6A—C5A—H5A	119.9	C4B—C5B—H5B	120.0
C4A—C5A—H5A	119.9	C6B—C5B—H5B	120.0
C5A—C6A—C7A	119.67 (17)	C5B—C6B—C7B	119.61 (19)
C5A—C6A—C9A	119.37 (17)	C5B—C6B—C9B	121.8 (2)
C7A—C6A—C9A	120.96 (18)	C7B—C6B—C9B	118.6 (2)
C8A—C7A—C6A	120.50 (18)	C8B—C7B—C6B	120.5 (2)
C8A—C7A—H7A	119.8	C8B—C7B—H6B	119.7
C6A—C7A—H7A	119.8	C6B—C7B—H6B	119.7
C7A—C8A—C3A	120.15 (17)	C7B—C8B—C3B	120.43 (19)
C7A—C8A—H8A	119.9	C7B—C8B—H7B	119.8
C3A—C8A—H8A	119.9	C3B—C8B—H7B	119.8
O1A—C9A—C6A	125.3 (2)	O1B—C9B—C6B	125.3 (3)
O1A—C9A—H9A	117.3	O1B—C9B—H9B	117.4
C6A—C9A—H9A	117.3	C6B—C9B—H9B	117.4

### Hydrogen-bond geometry (Å, º)

**Table:**

*D*—H···*A*	*D*—H	H···*A*	*D*···*A*	*D*—H···*A*
N1*A*—H1*NA*···N3*A*	0.84 (3)	2.32 (2)	2.630 (2)	102.0 (19)
N1*A*—H1*NA*···O1*A*^i^	0.84 (3)	2.41 (3)	3.190 (3)	154 (2)
N1*A*—H2*NA*···S1*A*^ii^	0.87 (3)	2.52 (3)	3.391 (2)	172 (2)
N2*A*—H3*NA*···S1*B*^iii^	0.84 (2)	2.50 (2)	3.3270 (19)	166.1 (19)
N1*B*—H1*NB*···N3*B*	0.91 (3)	2.21 (3)	2.619 (3)	106 (3)
N1*B*—H2*NB*···O1*B*^iv^	0.88 (3)	2.01 (3)	2.857 (3)	161 (3)
N2*B*—H3*NB*···S1*A*^v^	0.84 (2)	2.58 (2)	3.409 (2)	171 (2)

Symmetry codes: (i) −*x*+2, *y*+1/2, −*z*+1/2; (ii) −*x*+2, −*y*+2, −*z*; (iii) *x*, −*y*+3/2, *z*−1/2; (iv) *x*, *y*+1, *z*; (v) *x*, −*y*+3/2, *z*+1/2.

## References

[bb1] Bruker (2008). *SMART* and *SAINT* Bruker AXS Inc., Madison, Wisconsin, USA.

[bb2] Jagst, A., Sánchez, A., Vázquez-López, E. M. & Abram, U. (2005). *Inorg. Chem.* **44**, 5738–5744. 10.1021/ic050556t 16060625

[bb3] Lukmantara, A. Y., Kalinowski, D. S., Kumar, N. & Richardson, D. R. (2013). *Bioorg. Med. Chem. Lett.* **23**, 967–974. 10.1016/j.bmcl.2012.12.044 23312948

[bb4] Macrae, C. F., Bruno, I. J., Chisholm, J. A., Edgington, P. R., McCabe, P., Pidcock, E., Rodriguez-Monge, L., Taylor, R., van de Streek, J. & Wood, P. A. (2008). *J. Appl. Cryst.* **41**, 466–470.

[bb5] Serda, M., Mrozek-Wilczkiewicz, A., Jampilek, J., Pesko, M., Kralova, K., Vejsova, M., Musiol, R., Ratuszna, A. & Polanski, J. (2012). *Molecules*, **17**, 13483–13502. 10.3390/molecules171113483 PMC6268061 23151918

[bb8] Sheldrick, G. M. (1996). *SADABS* University of Göttingen, Germany.

[bb6] Sheldrick, G. M. (2008). *Acta Cryst.* A**64**, 112–122. 10.1107/S0108767307043930 18156677

[bb7] Westrip, S. P. (2010). *J. Appl. Cryst.* **43**, 920–925.

