# Supplementary figures and images for: Crystal structure of 1-(4-formyl­benzyl­idene)thio­semicarbazone
Source: Acta Crystallogr Sect E Struct Rep Online. 2014 Aug 6;70(Pt 9):o970. doi: 10.1107/S1600536814017255 (PMC4186181; doi:10.1107/S1600536814017255)

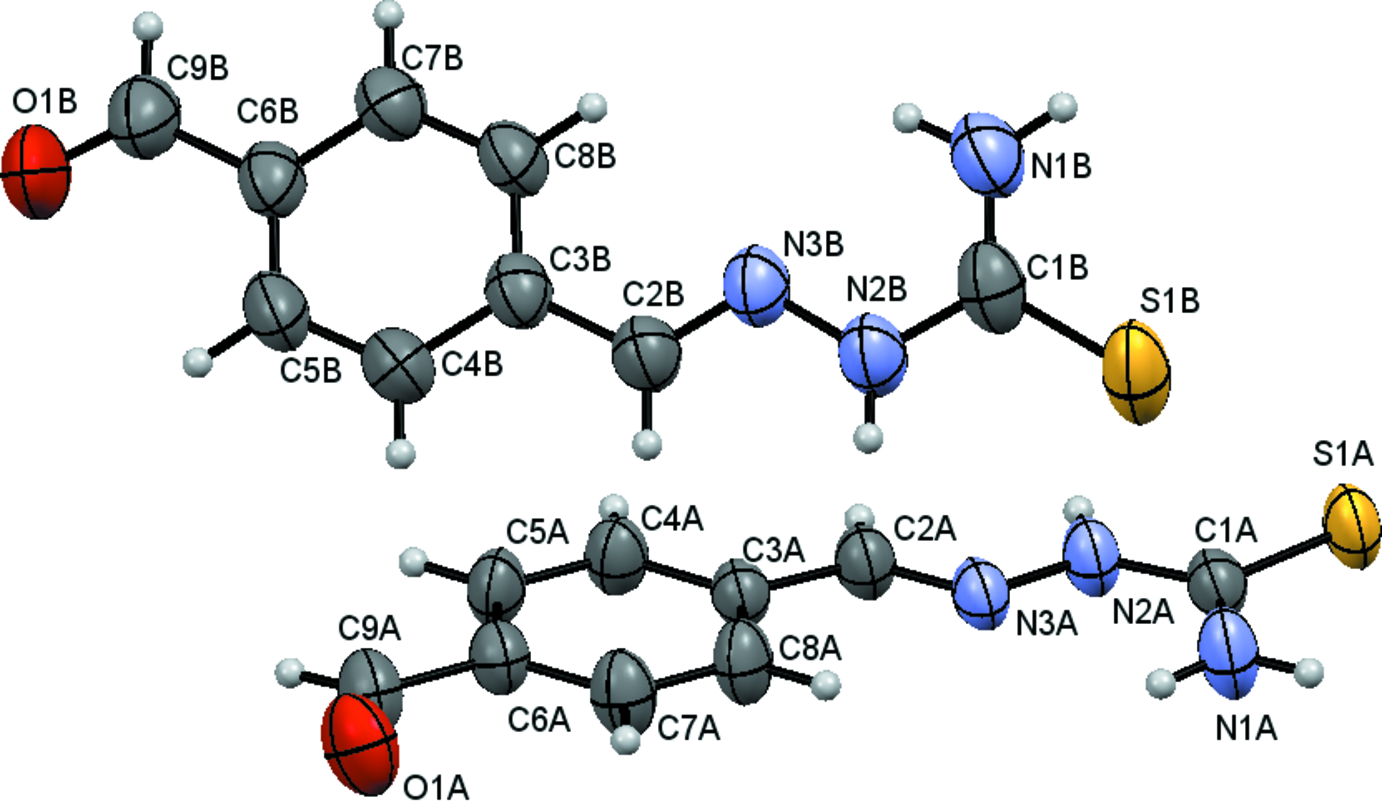

Supplement: Supplementary file 4 [file e-70-0o970-fig1.tif]

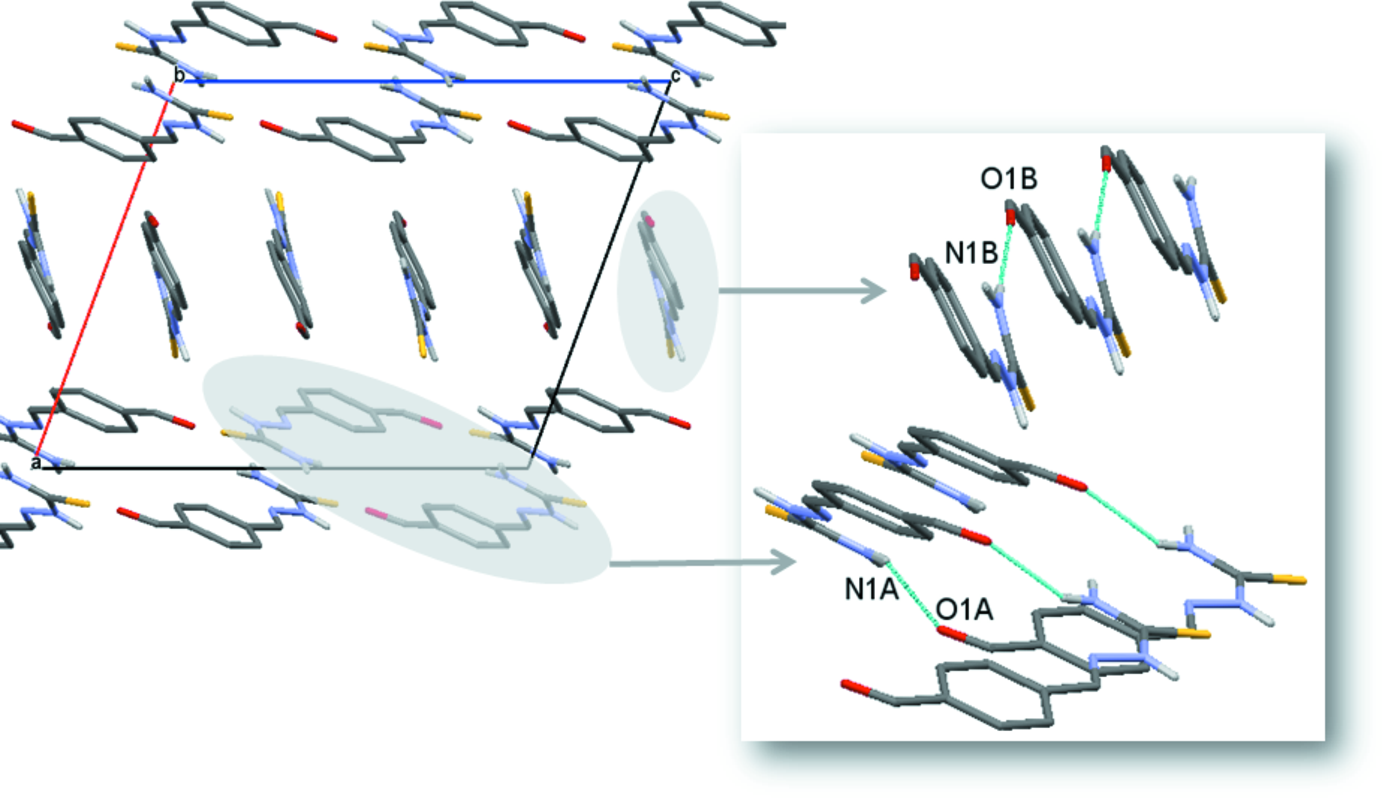

Supplement: Supplementary file 5 [file e-70-0o970-fig2.tif]
